# Low and high concentrations of butyrate regulate fat accumulation in chicken adipocytes via different mechanisms

**DOI:** 10.1080/21623945.2020.1738791

**Published:** 2020-03-12

**Authors:** Liqin Zhao, Shuang Liu, Zhihao Zhang, Jianmei Zhang, Xiaoqian Jin, Jing Zhang, Wenxiang Jiang, Haifang Li, Hai Lin

**Affiliations:** a College of Life Sciences, Shandong Agricultural University, Tai’an, China; b College of Animal Science and Veterinary Medicine, Shandong Agricultural University, Tai’an, China

**Keywords:** Sodium butyrate, fat accumulation, adipocytes, free fatty acid receptors (FFARs), histone deacetylase (HDAC) activity

## Abstract

The present study investigated the effects of varying concentrations of sodium butyrate (SB) on fat accumulation and cell proliferation in chicken adipocytes. High and low serial concentrations of SB used significantly reduced adipocytic fat accumulation. However, they were observed to exhibit differences in cell morphology and distinctions in lipogenic genes expression profiles. At lower concentration (0.01 mM), fat accumulation was decreased with an associated downregulation in the expression of lipogenic genes, which was mediated by free fatty acid receptors (FFARs). Contarily, at higher concentration (1 mM), the fat droplets laden in adipocytes were enlarged, and this was accompanied with activation of lipogenic genes expression. However, the total accumulated fat was also decreased largely due to reduction in cell numbers, which was partially attributable to the reduction in histone deacetylase (HDAC) activity. Animal experiments further indicated that dietary supplementation of lower dose coated SB (0.1% wt/wt) inhibited fat deposition in livers and abdominal fat tissues of broilers, suggesting the potential application of sodium butyrate as feed additive in the regulation of fat deposition.

## Introduction

Short-chain fatty acids (SCFAs) (mainly acetate, propionate and butyrate) are the major fermentation products produced by microorganisms in the caecum and colon using dietary fibres, such as food glue, resistant starch and indigestible carbohydrates [1–3]. The structure and abundance of microorganisms in the intestinal tract directly influence the production and proportion of SCFAs [1,4,5]. Mediation of the interaction between gut microbiota and host by SCFAs has been widely studied [1,2,4–6]. For instance, feeding rodents with dietary fibres inhibits body weight gain and fat accumulation due to changes in the gut microbiome and the produced SCFAs [1]. Sub-therapeutic administration of antibiotics increases adiposity in mice via changing the population structure of the gut microbiome and its metabolic ability to ferment carbohydrates to SCFAs [5]. Lowered abundance of universal butyrate- producing bacteria and a deficiency in SCFA production are associated with type 2 diabetes mellitus (T2DM) [6]. Dietary fibres could promote some SCFA-producing strains and elevate the production of acetate and butyrate, thus providing T2DM patients with improvements in glucose levels [6].

Among the three main SCFAs, the beneficial role of butyrate in health has been most studied in mammals [7–10]. For example, butyrate serves as a principal energy source for colonic epithelial cells and in protecting intestinal health [7]. It has the capacity to maintain body weight, insulin sensitivity and energy balance [8,9]. With regards to the role of butyrate in fat metabolism, inconsistent findings have been reported by different researchers. Gao *et al*. [8] demonstrated that dietary supplementation of sodium butyrate (SB) at 5% wt/wt in HFD could reduce fat accumulation in mice partially via enhancing fatty acid oxidation. In type 2 diabetic rats, SB administration (0.8 g/kg body weight/day) strongly suppressed fat deposition in livers [9]. Chen *et al*. [10] indicated that 0.5 µM SB could inhibit the adipogenic differentiation of human mesenchymal stem cells (MSCs). However, dietary supplementation of 1% SB for maternal mice was able to enhance ectopic fat deposition in the muscle of offsprings [11]. In the adipogenic differentiation of porcine stromal vascular fraction (SVF) cells, SB higher than 1 mM exhibited positive regulation on fat accumulation [12]. Collectively, the inconsistency of the above findings might be due to the differences in experimental models and the varied doses used in each experiment.

Lipid metabolism in chickens is different from that in mammals. In chickens, the liver is the primary site for fat synthesis, and the adipose tissue is mainly for fat deposition [13]. Therefore, there may exist differences in butyrate’s regulation of lipid metabolism between chickens and that in mammals. Some butyrate derived feed additives, such as SB, calcium butyrate, and butyrate glycerides have been used in poultry production [14–18]. Although some studies have identified a reduced abdominal fat ratio and an altered hepatic genes expression with dietary supplementation of butyrate derivatives in broiler chickens [17,18], the underlying mechanisms remain to be fully understood. Additionally, whether butyrate at different concentrations exert distinct effects on chicken fat accumulation is largely unknown.

In the present study, in *vitro* experiments were performed primarily to detect the effects of serial concentrations of SB on fat accumulation in chicken adipocytes. Secondly, the role of SB in cell proliferation was examined via EdU and CCK-8 assays in pre-adipocytes. Subsequently, the involvement of free fatty acid receptors (FFARs), extracellular regulated protein kinase (ERK) signalling, AMP-activated protein kinase (AMPK) signalling, and inhibition of histone deacetylase (HDAC) with low and high concentrations of SB was elucidated, respectively. Lastly, animal experiment was carried out to determine the influence of low dose butyrate (basal diets supplemented with 0.1% SB coated with polyacrylic resin П) on fat deposition in broiler chickens.

## Materials and methods

### Reagents

SB was purchased from Sigma-Aldrich (V900464, CA, USA). SB used in animal experiment was coated with polyacrylic resin II by Jiafa Granulation Drying Co., Ltd. (China). Bodipy 493/503 was purchased from Invitrogen (D3922, CA, USA). The iCLick Edu Andy FluorTM 488 imaging kit was purchased from GeneCopoeia (A003, Guangzhou, China). Trichostatin A (TSA) was from YEASEN (HB170410, Shanghai, China). Lipofectamine 2000 was obtained from Invitrogen (11,668–030, CA, USA). The HDAC activity colorimetric assay kit was from BioVision (K331-100, CA, USA). Synthetic double-stranded small interfering RNAs (siRNAs) were produced by Gene-Pharma (Shanghai, China). Antibodies to GAPDH, FABP4, and histone H3 were from Santa Cruz (CA, USA). Antibodies to AMPKα, phospho- AMPKα (Thr172), p44/42 MAPK (ERK1/2), phospho-p44/42 MAPK (ERK1/2) (Thr202/Tyr204), PPARG, and acetyl-histone H3 (Lys9) were obtained from Cell Signalling (MA, USA).

### Isolation and culture of chicken preadipocytes

Primary chicken preadipocytes were isolated and cultured as described previously [19]. Briefly, the adipose tissues from 17-day-old chicken embryos were minced, digested, filtered and centrifuged to remove other cell types. Subsequently, the preadipocytes were resuspended in DMEM medium containing 10% foetal bovine serum (FBS) and 1% antibiotic mixture. The cells were seeded into plates and cultured in a humidified atmosphere with 5% CO2 at 37°C until reaching subconfluence. To induce maturation of the preadipocytes, adipogenic cocktail stimuli (AS) was administered. The components of AS were as follows. Medium I (0_2 d): 5 μg/ml of porcine insulin, 1 μM dexamethasone, 1 μM rosiglitazone, 0.5 mM IBMX, and 10% FBS; Medium II (2_4 d): 5 μg/ml of porcine insulin, 1 μM dexamethasone, 1 μM rosiglitazone, and 10% FBS; Medium III (4_6 d): 5 μg/ml of porcine insulin, 1 μM rosiglitazone, and 10% FBS. Medium IV (6_8 d): 0.5 μg/ml of porcine insulin.

### Cell treatments

SB ranging from 0.01 to 2 mM were supplemented into cells during the induction period of preadipocytes into mature adipocytes. The concentrations were selected based on previous reports and the physiological ranges [10,12,20]. To detect the phosphorylation status of ERK and AMPK, confluent preadipocytes were treated with 0.01 mM SB or 1 mM SB for 3 min, 5 min, 10 min, 30 min, and 60 min, respectively. To determine the involvement of HDAC inhibition in butyrate effect on adipocytes, HDAC activity was examined after treating the cells with SB for 4 days in the presence of AS. Histone H3 and acetyl-histone H3 protein levels were detected at day 8 post treatment. TSA (a cell-permeable, highly selective inhibitor of HDACs) was used to mimic the effect of butyrate on adipogenic differentiation.

### Cell transfections

To downregulate FFAR expression, specific siRNAs were transfected into the cells. Preadipocytes were cultured in an antibiotic-free medium for 24 h. Then, 120 nM of total siRNA and 3.0 μL of Lipofectamine 2000 were diluted in separate tubes in Opti_MEM™ (Gibco, CA) and incubated for 15 min at room temperature (RT). The two solutions were mixed and incubated for another 30 min at RT to form transfection complexes. After 8 h, the Opti_MEM was replaced with DMEM/10% FBS, and SB was added to the culture medium. The cells were incubated at 37°C/5% CO2 and harvested at indicated days post-transfection. The sequences of the specific siRNAs are listed in Table 1.

**Table 1.** The primers and siRNAs used in this study

Genes	Sequence 5ʹto 3ʹ
GAPDH	Forward-CTACACACGGACACTTCAAG Reverse-ACAAACATGGGGGCATCAG
PPARG	Forward-AGACACCCTTTCACCAGCATCC Reverse-AACCCTTACAACCTTCACAAGCA
FAS	Forward-TCCTTGGTGTTCGTGACG Reverse-CGCAGTTTGTTGATGGTGAG
ADPN	Forward-TCACCTACGACCAGTTCCA Reverse-CCCGTTGTTGTTGCCCTC
FABP4	Forward-TGAAGCAGGTGCAGAAGT Reverse-CAGTCCCACATGAAGACG
LPL	Forward-CAGTGCAACTTCAACCATACCA Reverse-AACCAGCCAGTCCACAACAA
UCP-3	Forward- GCAGCGGCAGATGAGCTT Reverse-AGAGCTGCTTCACAGAGTCGTAGA
FFAR2	Forward-AACGCCAACCTCAACAAGTC Reverse-TGGGAGAAGTCATCGTAGCA
FFAR3	Forward-GAAGGTGGTTTGGGAGTGAA Reverse-CAGAGGATTTGAGGCTGGAG
ACC	Forward-AATGGCAGCTTTGGAGGTGT Reverse-TCTGTTTGGGTGGGAGGTG
SREBP-1 c	Forward-GCCCTCTGTGCCTTTGTCTTC Reverse–ACTCAGCCATGATGCTTCTTCC
SiRNA-FFAR2	Forward-GCUUCUUCUCCAGCAUCUATT Reverse-UAGAUGCUGGAGAAGAAGCTT
SiRNA-FFAR3	Forward-CCCACUGUUCCAUCAUCUUTT Reverse-AAGAUGAUGGAACAGUGGGTT

### EdU and CCK-8 assays

EdU, which is a rather new thymidine analog that can be incorporated into DNA during active DNA synthesis, has widely been used to trace the cell proliferation ability [21]. In this study, subconfluent preadipocytes were treated with different SB doses for 24 h. Then, the cells were incubated with 10 µM EdU for 2 h. Cell increment could be detected via a chemical reaction with Andy FluorTM 488 Azide (green fluorescence). The nuclei were stained with Hoechst 33,342 (blue fluorescence). Pictures were photographed with a two-photon laser confocal microscope (Zeiss, Germany).

CCK-8 analysis was conducted to examine the cell viability. After treating subconfluent preadipocytes for 24 h with serial concentrations of SB, CCK-8 solution (Vazyme, Nanjing) was added to the cells. Following incubation with CCK-8 for 3 h, the absorbance at 450 nm was measured with a microplate reader (BioTek, USA).

### Bodipy staining and oil red O staining

On d 8 post-induction, adipocytes grown on slides were washed twice with D-Hank’s and subsequently fixed with 4% paraformaldehyde for 1 h at RT. Adipocytes were stained with bodipy (1 µg/mL) for 30 min and then with DAPI (1 µg/mL) for 5 min. The cultures were photographed with a two-photon laser confocal microscope (Zeiss, Germany).

The differentiated adipocytes were stained with oil red O on d 8 post-treatment. Cells were washed twice with D-Hank’s and subsequently fixed with 4% paraformaldehyde for 1 h at RT. Following fixation, the cells were washed twice with D-Hank’s and subsequently stained with 0.6% oil red O solution for 1 h. Haematoxylin staining was performed to visualize the cell nuclei. After washing, the cultures were photographed with an inverted microscope (Olympus, Japan). The stained oil red O was quantified and expressed as mmol/g total protein.

### Determination of triglyceride (TG) contents in adipocytes

The differentiated adipocytes were lysed by RIPA Lysis Buffer. TG contents in the lysed extracts were analysed using a commercial assay kit (Nanjing Jiancheng, China), and expressed as mmol/g total protein.

### Total RNA extraction and qRT-PCR

Total RNA was extracted from treated cells or tissues, and qRT-PCR was performed as described previously [13]. The first strand cDNAs were synthesized, and quantitative measurements were performed with SYBR Green I labelling (Roche, USA). Real-time PCR was performed at 95°C for 10 s, followed by 40 cycles of 95°C for 5 s and 60°C for 40 s. The PCR data were analysed with the 2^−∆∆Ct^ method using GAPDH as the reference gene. Agarose-gel electrophoresis and DNA sequencing were conducted to verify the specificity of the amplified products. The primer sets for the related genes are listed in Table 1.

### Western blotting

Treated cells were lysed in RIPA Lysis Buffer supplemented with protease or phosphatase inhibitors. The cell lysates were centrifuged at 12,000 × g for 15 min at 4°C, and the supernatants were collected. Equal amounts of proteins were separated by SDS-PAGE and transferred onto nitrocellulose membranes. Immunoblotting was performed using primary antibodies for proteins of interest, followed by HRP-conjugated secondary antibodies. The protein signals were detected using ECL Plus (Beyotime, Shanghai).

### HDAC activity determination

Nucleoproteins were extracted from control or SB-treated cells. Samples were diluted to the desired protein concentration. Total HDAC activity was measured using the HDAC Activity Colorimetric Assay Kit (BioVision, CA). The HDAC colorimetric substrate, which comprises an acetylated lysine side chain, was incubated with the protein samples for 30 min at 37°C. Deacetylation sensitizes the substrate, and the lysine developer produces a chromophore, which can be easily analysed using a microplate reader at 405 nm.

### Animals and experimental protocol

Animal experiment was conducted at the research farm of Shandong Agricultural University. All experimental procedures were approved by the Animal Care and Use Committee of Shandong Agricultural University. A completely randomized design consisting of 2 dietary treatments was used: (1) Control: a basal diet supplemented with polyacrylic acid resin II (the coating material). (2) SB: a basal diet supplemented with 0.1% sodium butyrate coated with polyacrylic acid resin II (70% of sodium butyrate was protected). The ingredients and the analysed nutrient compositions of the basal diet are provided in Supplementary Table 1.

A total of 120 healthy 1-day-old male broilers (Arbour Acres) were used. Each of the 2 diets was fed to 6 replicates of 10 birds each. All birds had free access to food and water during the rearing period. The feed intake and body weight were assessed at d 21 and d 42, respectively. Blood sample**s** were obtained from the wing veins. Serum was collected after centrifugation at 3500 × *g* for 10 min and stored at – 80°C prior to analysis. The broilers were sacrificed after anaesthetization, and tissue samples (abdominal fat and liver) were collected. The liver index and abdominal fat rate were calculated based on tissue weight/body weight %. TG and TCH contents in serum and liver tissues were determined by commercial detection kits from Nanjing Jiancheng (China). Also, abdominal fat and liver tissues were used for RNA extraction and qRT-PCR analysis for adipogenic genes expression.

### Histological analysis

The hepatic and abdominal fat tissues were fixed in 4% paraformaldehyde, and histological slides were prepared as described previously [9]. The paraffin sections were deparaffinized with xylene and rehydrated with alcohol and water. The slides were stained with haematoxylin and eosin (H&E), and photographed under a microscope (Olympus, Japan). Area with adipocytes in adipose tissues were measured by using an image software (Nikon, Japan).

### Statistical analysis

Results were analysed using the SAS statistical software (SAS version 8e, SAS Institute, 1998). Data were presented as means ± standard error of mean (SEM). Significant difference between treatments was tested by one-way ANOVA or two-sample student *t*-test. Differences were considered significant when *p* < 0.05.

## Results

### Low and high concentrations of SB regulate fat accumulation in chicken adipocytes in different manners

**Figure 1.**
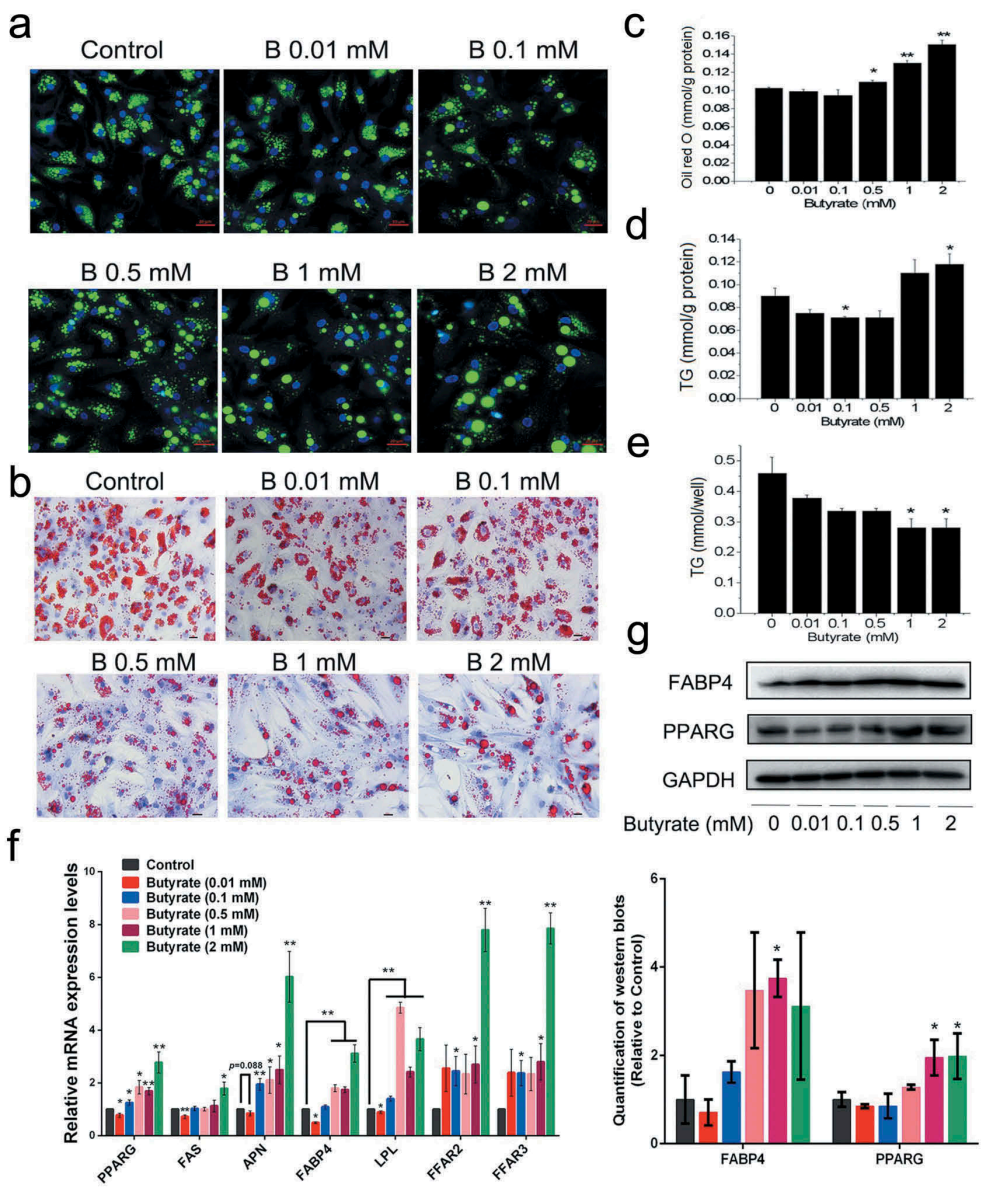
**Effect of serial concentrations of sodium butyrate (SB) on fat accumulation in chicken adipocytes**. Chicken preadipocytes were incubated for 8 days with 0.01, 0.1, 0.5, 1, or 2 mM SB in the presence of adipogenic stimuli (AS). (a) Lipid droplets visualized by confocal microscopy in differentiated adipocytes upon bodipy (green)-staining, colocalizing with DAPI (blue)-stained nuclei. Scale bars represent 100 μm. (b) Lipid droplets visualized in differentiated adipocytes upon oil red O (red)-staining. The nuclei were stained with haematoxylin (purple). Scale bars represent 100 px. (c) The stained oil red O was quantified after isopropanol extraction, which was shown as mmol/g total protein. (d) The TG contents from differentiated adipocytes upon different treatments, which were shown as mmol/g total protein. (e) The pure TG contents in differentiated adipocytes upon different treatments, which were shown as mmol/well. (f) Relative mRNA levels of lipogenic markers and FFARs determined by qRT-PCR in the treated cells on day 8. The mRNA levels were normalized to GAPDH. (g) Representative images of western blots and quantitative analysis of the expression of lipogenic markers on day 8 post treatment (n = 3). GAPDH serves as a loading control. Data are the means ± SEM of at least 3 independent experiments. *p < 0.05, **p < 0.01 *vs*. the control (0 mM SB)

Preadipocytes were treated with SB ranging from 0.01 to 2 mM during the adipogenic process. Bodipy staining showed that the 0.01 and 0.1 mM butyrate groups had fewer stained lipid droplets than the control and that droplet sizes did not change significantly. However, SB at or higher than 0.5 mM resulted in larger lipid droplets and reduced cell numbers, especially at the 1 mM and 2 mM concentrations (Figure 1(a)). Oil red O staining results showed a similar tendency as the bodipy staining (Figure 1(b)). Likewise, it was observed that based on same protein content, butyrate at or lower than 0.5 mM reduced fat accumulation, but at concentrations higher than 1 mM, fat accumulation in adipocytes were increased significantly (Figure 1(c,d)). However, the total TG content per well drastically reduced with butyrate concentrations in a dose-dependent manner (Figure 1(e)).

SB activated FFAR2 and FFAR3 mRNA expression, with 2 mM showing the highest expression compared to other groups (Figure 1(f)). The 0.01 mM SB treated group, had the PPARG (peroxisome proliferators-activated receptor γ), FABP4 (fatty acid binding protein 4), FAS (fatty acid synthase) and LPL (lipoprotein lipase) mRNA levels statistically lower than those in the controls. Contrarily, SB-treated groups higher than 0.5 mM showed marked elevated expressions of all adipogenic genes tested (Figure 1(f)). Western blotting revealed that adipocytes treated with 1 mM or 2 mM butyrate had upregulated protein expression of PPARG and FABP4 compared with untreated cells (Figure 1(g)). These findings indicate the differential response of butyrate treated adipocytes at lower or higher concentrations on fat accumulation in *vitro*.

### SB affects the proliferation of chicken preadipocytes

**Figure 2.**
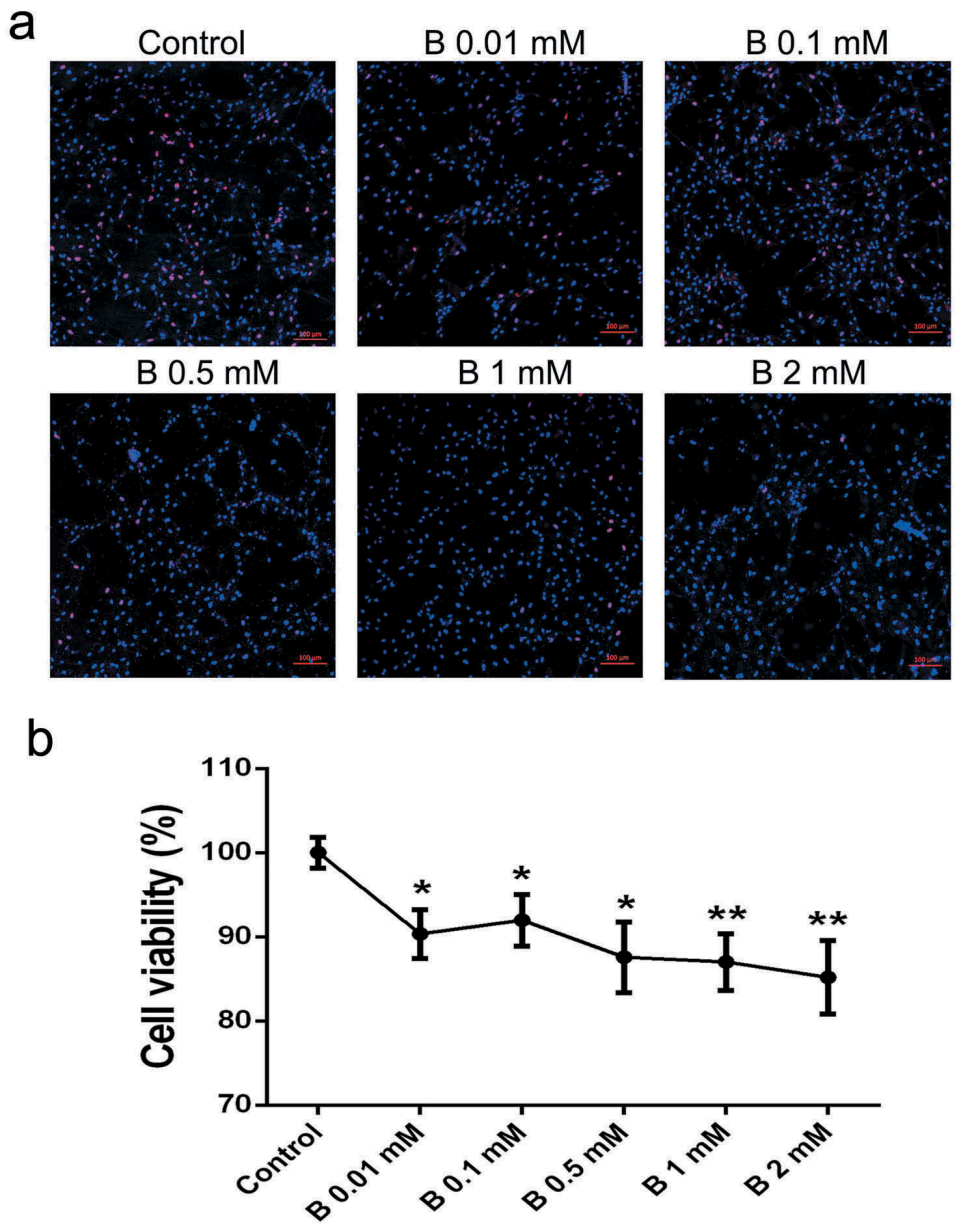
**SB inhibits preadipocyte proliferation**. (a) EdU assay was performed after the cells were incubated for 24 h with serial concentrations of SB. Confocal microscopy of preadipocytes perfused with EdU (red) and counterstained with Hoechst 33,342 (blue). The EdU/Hoechst 33,342 ratio represents the cell proliferation rate. Scale bars represent 100 μm. (b) CCK-8 assay was conducted to detect cell viability after butyrate treatment. The data are the means ± SEM (n = 6). *p < 0.05, **p < 0.01 *vs*. the control (0 mM SB)

During cell cultivation, we found SB had the capacity to suppress cell growth, especially at higher concentrations. Therefore, serial concentrations of SB (0.01, 0.1, 0.5, 1 and 2 mM) were used to determine their effects on preadipocyte proliferation. EdU assay can trace the proliferation ability of cells. The results revealed that butyrate at concentrations of 0.5 mM or higher, can significantly reduce the percentage of EdU-positive cells compared to controls (Figure 2(a)). Moreover, cell viability measured by the CCK-8 assay showed that butyrate inhibited cell growth in a dose-dependent manner. Cell viability was down-regulated by 9.2%, 8.0%, 12.4%, 13.0%, and 14.8% in SB treated cells ranging from 0.01 to 2 mM, respectively (Figure 2(b)).

### Signalling pathways involved in low SB dose-induced effects on fat accumulation in adipocytes

**Figure 3.**
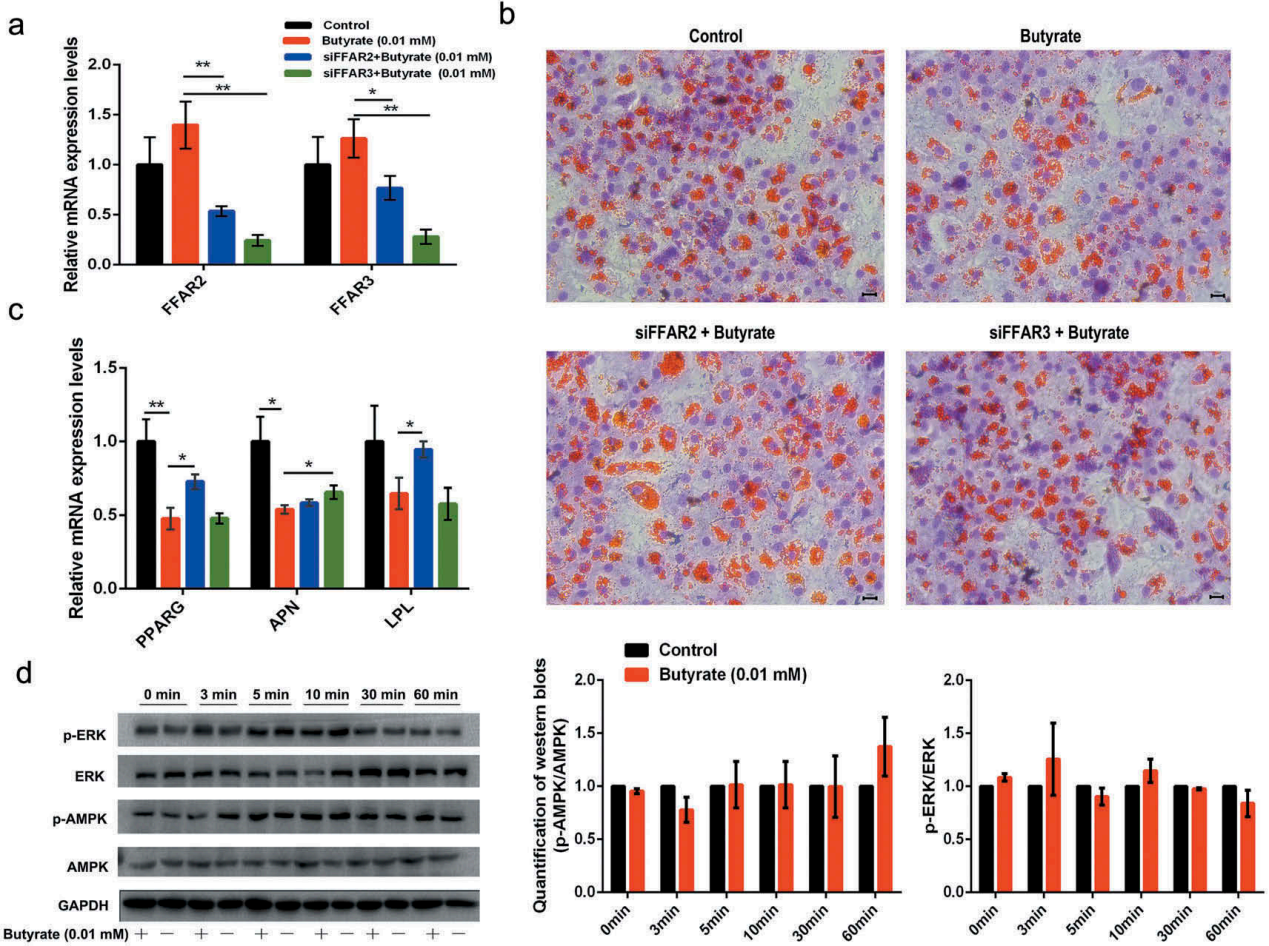
Signalling elucidation of the role of 0.01 mM SB in fat accumulation

Following previous findings, 0.01 mM and 1 mM concentrations were selected to study the signalling pathways underlying the distinct effects of SB on fat accumulation. Since butyrate at all concentrations increased FFAR2 and FFAR3 mRNA expression, siRNAs specific to the two receptors were used to down-regulate their expression. The result in Figure 3(a) showed that the transcription of both receptors was significantly inhibited by siRNA-FFAR2 and siRNA-FFAR3, respectively. Oil red O staining revealed that siRNA-FFARs reversed the lessened fat droplets under 0.01 mM SB treatment (Figure 3(b)). As shown in Figure 3(c), 0.01 mM SB extensively decreased the mRNA levels of PPARG, APN (adiponectin), and LPL. siRNA-FFAR2 recovered the 0.01 mM SB-suppressed PPARG and LPL mRNA levels by 52.3% and 46.0%, respectively. However, siRNA-FFAR3 only rescued the down-regulated APN mRNA level by 21.7%.

(a)The effect of specific siRNAs on the mRNA expression of FFAR2 and FFAR3, determined by qRT-PCR. (b) Oil red O staining was conducted in differentiated adipocytes treated with 0.01 mM SB alone or together with siRNA-FFARs. The nuclei were stained with haematoxylin (purple). (c) Relative mRNA levels of lipogenic markers were determined by qRT-PCR in the treated cells on day 8. (d) Representative images of western blots and quantitative analysis of the expression of p-ERK, ERK, p-AMPK and AMPK after the cells treated with 0.01 mM SB or vehicle (n = 3). GAPDH serves as a loading control. The data are the means ± SEM (n = 3_6). * *p* < 0.05, ** *p* < 0.01 *vs*. the control (0 mM SB).

**Figure 4.**
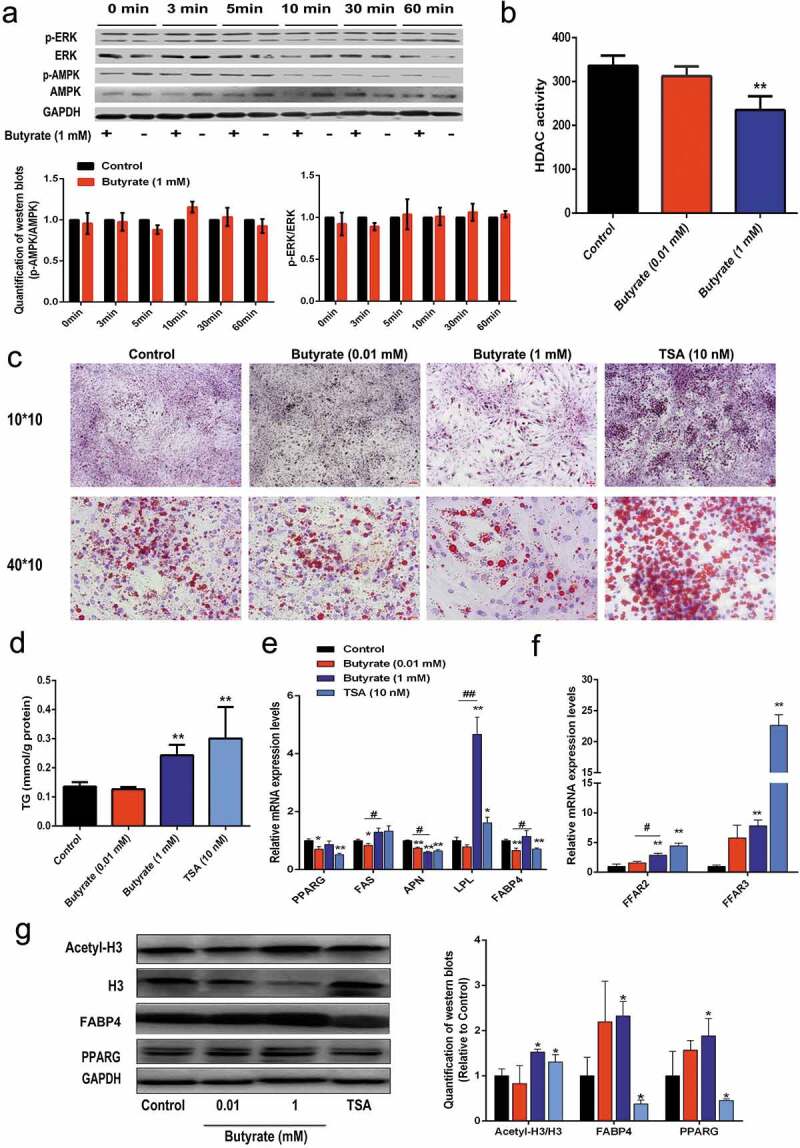
Signalling elucidation of the role of 1 mM SB in fat accumulation

ERK is one of the major signalling pathways that affects cell growth. Since SB was able to inhibit cell proliferation, its effect on ERK signalling was eveluated. However, 0.01 mM SB did not change the phosphorylation status of ERK, with similar p-ERK/ERK ratios during the 60 min intervals (Figure 3(d)). AMPK is considered as a cellular energy sensor that regulates lipid metabolism by phosphorylating key regulatory enzymes. Therefore, alteration of AMPK phosphorylation was examined in the presence of 0.01 mM SB. However, the p-AMPK/AMPK ratios were not altered by 0.01 mM SB at either time points (Figure 3(d)). In addition, 0.01 mM SB showed no obvious HDAC inhibition activity (Figure 4(b)). These data hint that 0.01 mM SB inhibits adipocyte formation in part through FFARs actions.

(a)Effect of 1 mM SB on phosphorylation of ERK and AMPK at the indicated time points, determined by western blotting and quantification analysis. GAPDH serves as a loading control. This experiment was repeated 3 times. (b) HDAC activity in the treated cells, determined using a commercial assay kit. **(c)** Oil red O staining (red) was performed in differentiated adipocytes treated with SB or TSA for 8 days. The nuclei were stained with haematoxylin (purple). (d) Quantification of the accumulated TG based on the same protein content. (e) Relative mRNA expression levels of PPARG, FAS, APN, LPL and FABP4 in treated cells. (f) Relative mRNA expression levels of FFAR2 and FFAR3 in treated cells. (g) Representative images of western blots and quantitative analysis of PPARG, FABP4, histone H3 and acetyl-histone H3 in the treated cells (n = 3). GAPDH was used as an internal control. The data are the means ± SEM (n = 3_6). *p < 0.05, ** *p* < 0.01 *vs*. the control; # *p* < 0.05, ## *p* < 0.01 indicate differences between the 0.01 mM and 1 mM treatments.

### Signalling pathways involved in the role of high SB dose-induced effects on fat accumulation in adipocytes

To elucidate the signalling pathways involved in the role of 1 mM SB in fat accumulation, its effects on ERK signalling, AMPK signalling, and HDAC activity were determined. Results in Figure 4(a) indicated that neither the p-ERK/ERK nor the p-AMPK/AMPK ratio was markedly altered by 1 mM SB. Strikingly, 1 mM SB significantly inhibited HDAC activity (Figure 4(b)). Therefore, TSA, which is a highly specific inhibitor of HDACs, was used to mimic the effect of SB. The acetyl-histone H3 protein expression level was elevated by the 1 mM SB and 10 nM TSA treatments, respectively (Figure 4(g)). Oil red O staining and TG content determination showed that both 1 mM SB and 10 nM TSA groups accumulated more lipids than the control group (Figure 4(c,d)). However, different from 1 mM SB, 10 nM TSA did not significantly reduce the cell number (Figure 4(c)). Similar to the effects of 1 mM SB, 10 nM TSA also vastly elevated FFAR expression, with about 4-fold and 22-fold increase in FFAR2 and FFAR3 mRNA expressions, respectively (Figure 4(f)). Moreover, 1 mM SB and 10 nM TSA exhibited some increment in the FAS and LPL mRNA levels, whereas the two treatments displayed an opposite tendency on the mRNA and protein expression of PPARG and FABP4 (Figure 4(e,g)). These findings demonstrate that inhibition of HDAC activity is at least partially involved in the role of 1 mM SB in fat accumulation.

### Dietary SB supplementation reduces fat deposition in broilers

**Figure 5.**
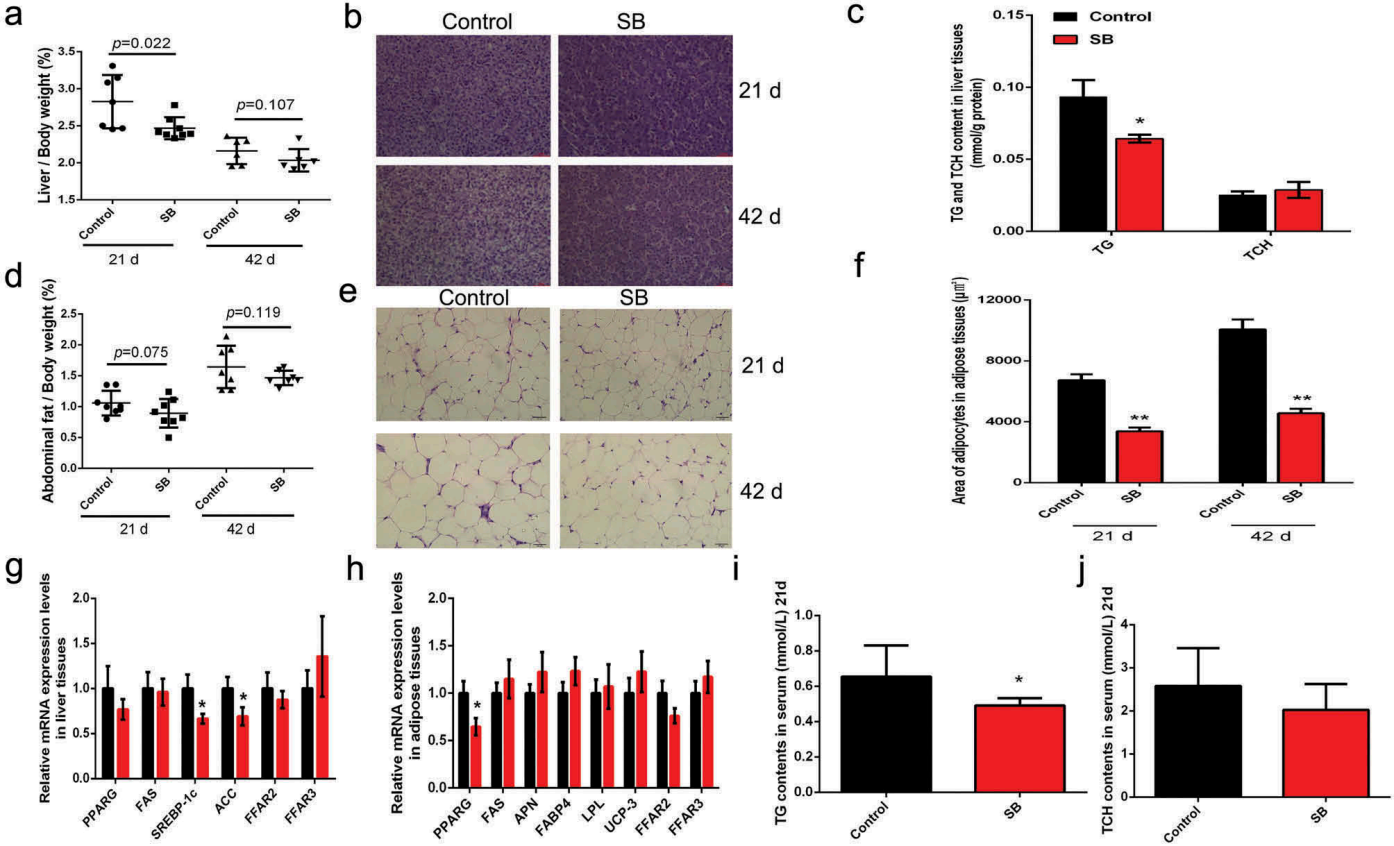
Dietary SB supplementation (0.1%) reduces fat deposition in broilers

Liver and abdominal fat samples were collected at d 21 and d 42, and the liver indexes and abdominal fat ratios were calculated. Liver index was reduced in SB-treated animals at d 21 (Figure 5(a)). H&E staining showed that fat deposition in the livers was decreased with SB supplementation (Figure 5(b)). In accordance, the TG content in SB-treated livers was significantly lower than that in control livers (Figure 5(c)). In contrast to the controls, SB supplementation substantially although not significantly reduced the abdominal fat ratio at both d 21 and d 42, which was decreased by 16.5% and 17.6%, respectively (Figure 5(d)). H&E staining and adipocyte area measurement indicated that SB treatment extensively decreased the size of the abdominal fat cells compared to control groups (Figure 5(e,f)). As shown in Figure 5(g,h), the transcription of some adipogenic genes (Srebp-1 c and ACC in liver; PPARG in adipose tissue) was inhibited in SB-treated livers and adipose tissues. The serum TG level from SB-treated broilers was significantly decreased compared to that from the control animals (Figure 5(i)). However, the growth of the broilers and the feed to gain ratio were not changed by SB administration (Supplementary Figure 1). These findings indicate that fat deposition in broilers was reduced by dietary supplementation of low dose SB.

(a) Liver indexes in the control- and SB-treated broilers. (b) H&E staining of the liver slides at d 21 and d 42. Scale bars represent 50 μm. (c) TG and TCH contents in liver tissues at d 21. (d) Abdominal fat ratios in the control- and SB-treated broilers. (e) H&E staining of the abdominal fat slides at d 21 and d 42. Images are representative pictures for each treatment. scale bars represent 50 μm. (f) Quntification of the adipocyte area in abdominal fat tissues. (g) The relative mRNA levels of fat synthesis associating genes and FFARs in liver tissues of 21-day-old broilers. (h) The relative mRNA levels of fat deposition associating genes and FFARs in adipose tissues of 21-day-old broilers. (i) Serum TG content in the control- and SB-treated broilers at d 21. (j) Serum TCH content in the control- and SB-treated broilers at d 21. The data are the means ± SEM of n = 5_8. *p < 0.05 *vs*. the control.

## Discussion

The main findings of this study are as follows: 1) SB at low concentrations inhibited adipocytic fat accumulation through activating FFAR2 and FFAR3; 2) SB at high concentrations showed HDAC inhibition activity, which partially resulted in larger fat droplets in adipocytes; 3) SB had the capacity to inhibit cell proliferation in a dose-dependent manner; 4) dietary supplementation of low dose coated SB suppressed both hepatocytic and adipocytic fat deposition in broilers.

Although large scale studies have demonstrated the regulation of butyrate on fat accumulation [9,10,12,17], the difference associating with different concentrations was not clearly defined. We found here that, though butyrate ranging from 0.01 mM to 2 mM could reduce fat accumulation in chicken adipocytes, the cell morphology and the underlying mechanisms were profoundly different between low and high concentrations. To elucidate whether lipolysis contributes to the SB-reduced fat deposition in chicken adipocytes, we treated the differentiation adipocytes with varying concentrations of SB for 48 h. Results in Supplementary Figure 2(a) showed that SB higher than 0.5 mM increased the mRNA levels of adipose triglyceride lipase (ATGL), while only 2 mM SB elevated the transcription of carnitine palmitoyl transterase-1 (CPT-1). Meanwhile, the droplets in high dose SB-treated adipocytes were still larger than the controls (Supplementary Figure 2(b)). These data hinted that SB reduced adipocytic fat accumulation majorly via inhibiting fat formation rather than via enhancing fat mobilization. In view of previous studies performed in mammals, most butyrate produced in the intestine is absorbed by intestinal epithelial cells as energy. A small fraction functions as a signal molecule, which is transported to other target tissues through the blood circulation. Cells in target tissues mainly receive the signals through FFAR3 [22,23]. Thereafter, the signals affect downstream signalling pathways related to fat synthesis, fat mobilization, or fatty acid oxidation [3,5,8]. Thus, we first examined the influence of butyrate on the expression of FFARs in chicken adipocytes. Surprisingly, both FFAR3 and FFAR2 were elevated by SB in a sensitive manner, even at lower doses. When the two receptors were blunted by specific siRNAs, the low SB dose-induced inhibition on fat formation was reversed. These findings indicated that both FFAR3 and FFAR2 could receive butyrate signals and that the two receptors at least partially mediated the effect of low dose butyrate on fat accumulation in chicken adipocytes. This was inconsistent with the findings from mammals, in which adipocytes received butyrate signals mianly through FFAR3 [22].

It has been demonstrated that butyrate can regulate the acetylation level of lipid-related genes through its HDAC inhibition activity [9,12]. Thus, we tested the influence of SB on HDAC activity. Notably, only the high dose SB could reduce HDAC activity. Similar to TSA (a highly specific HDAC inhibitor), SB at high dose significantly enhanced the acetyl-histone H3 levels. Additionally, TSA can partially mimic the effect of high dose butyrate on fat accumulation. AMPK phosphorylation and the MAPK (mitogen-activated protein kinase) pathway have been reported to be involved in the role of butyrate in fat metabolism in mammals [8–10,20]. However, in this study, neither the p-AMPK/AMPK nor the p-ERK/ERK ratio was affected by butyrate at either low or high dose. Thus these findings indicate that butyrate at high dose affected fat accumulation at least partially via both FFARs and its HDAC inhibitory activity.

It should be noted that, although SB at higher doses led to larger lipid droplets, they simultaneously lowered the cell proliferation rate. The concentrations we used did not change the pH (data not shown), hinting that the inhibition of SB on cell proliferation was not caused by pH alteration. Inhibition of butyrate on HDAC activity has been well characterized in various cancerous and noncancerous disorders, in which butyrate can decrease the cell growth and proliferation rates [7,24]. Thus, the suppression of SB on cell proliferation observed in this study might be as a result of its HDAC inhibitory activity.

Most previous reports have demonstrated negative modulation of fat accumulation by butyrate in mammals, with the adipose tissue as the major organ of concern [8–10]. Chicken represents a unique case of lipid metabolism that mainly synthesizes in liver and deposits in fat tissue [13]. We found that both chicken adipose tissues and livers expressed FFAR2 and FFAR3, with adipose tissues owning higher expression levels than the livers (Supplementary Figure 3). Thus, we primarily examined the effects of varied concentrations of SB on fat accumulation in adipocytes. Notably, a similar effect of SB on fat deposition was also observed in chicken hepatocytes, in which SB showed inhibitory effects on fat deposition at both lower or higher doses, although higher doses exhibited larger droplets and less cell numbers (Supplementary Figure 4). These results provide evidence for butyrate involvement in fat accumulation within both chicken adipocytes and hepatocytes.

Since SB can be used as feed additive at lower dosage in chicken breeding, the effect of 0.1% coated SB on chicken fat deposition was studied. The purpose of coating with polyacrylic resin II was to allow a slow and gradual release of SB within the digestive tract. As expected, the butyrate contents in caecal chyme were indeed increased although not statistically in the SB-treated broilers (Supplementary Figure 5). Results from animal experiment indicated that the liver index, abdominal fat ratio, lipid deposition in the livers and in fat tissues were all lowered moderately to certain extents in SB-treated broilers. Similarly, the serum TG content was also decreased in the SB treated groups. Notably, although the fat deposition was reduced, the growth performance and feed to gain ratio of broilers were not changed by SB administration (Supplementary Figure 1). These findings indicate that low dose SB suppressed fat deposition in broilers by inhibiting fat synthesis in livers and fat accumulation in adipose tissues. This could ensure the promising application of SB in chicken breeding.

As a feed additive, SB is prone to alter the structure of gut microbiome [3,4,14]. Our data provides evidence that SB could change the intestinal microbial flora of broilers (Supplementary Figure 6). One explanation for alteration of the gut microbiota by butyrate may be its effect on the gut micro environment, such as down regulation of the pH. pH lowering enhances the growth of some acid-resistant bacteria, such as *Lactobacillus* and *Coprococcus* [25,26] (Supplementary Figure 6), and these bacteria become increasingly dominant. Many of these acid-resistant bacteria have the capacity to produce some type of SCFAs, which changes the production and finally the proportion of SCFAs (Supplementary Figure 5). Meanwhile, the dominant growth of these beneficial bacteria reduces some harmful or acid-sensitive bacteria, similar to the reduction of *Helicobacter, Campylobacter*, and *Bifidobacterium* observed in this study [27,28] (Supplementary Figure 6).

Collectively, this study demonstrates a novel understanding on the differential responses of lower and higher doses of butyrate on fat accumulation in chicken adipocytes (Supplementary Figure 7). Our findings also provided a theoretical basis for controlling fat deposition by using low dose butyrate in chicken breeding.

## Supplementary Material

Supplemental Material
