# Supplementary material for: Low and high concentrations of butyrate regulate fat accumulation in chicken adipocytes via different mechanisms
Source: Adipocyte. 2020 Mar 12;9(1):120–31. doi: 10.1080/21623945.2020.1738791 (PMC7153540; doi:10.1080/21623945.2020.1738791)
Supplement: Supplemental Material [file KADI_A_1738791_SM8524.docx]

**Supplementary files**


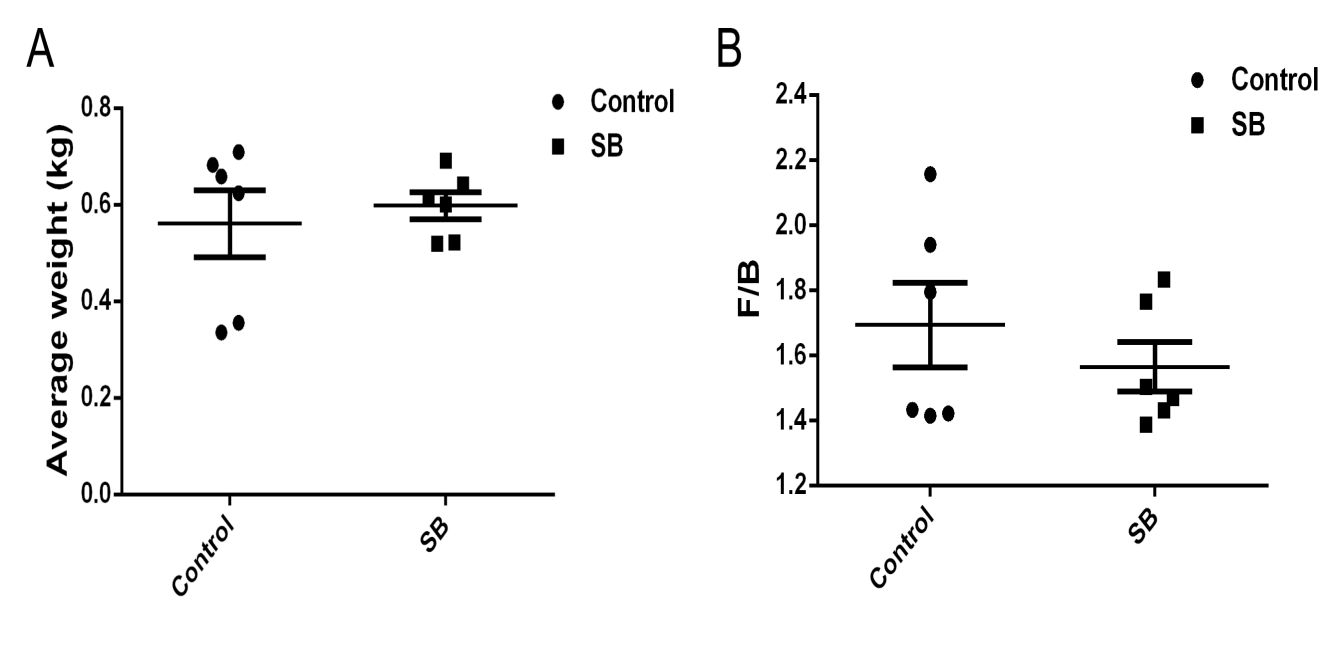


**Supplementary Figure 1. Dietary SB does not change the average weight (A) and the feed to gain (F/G) ratio (B).** Each black dot represents an independent sample. The data are the means ± SEM (n=6).


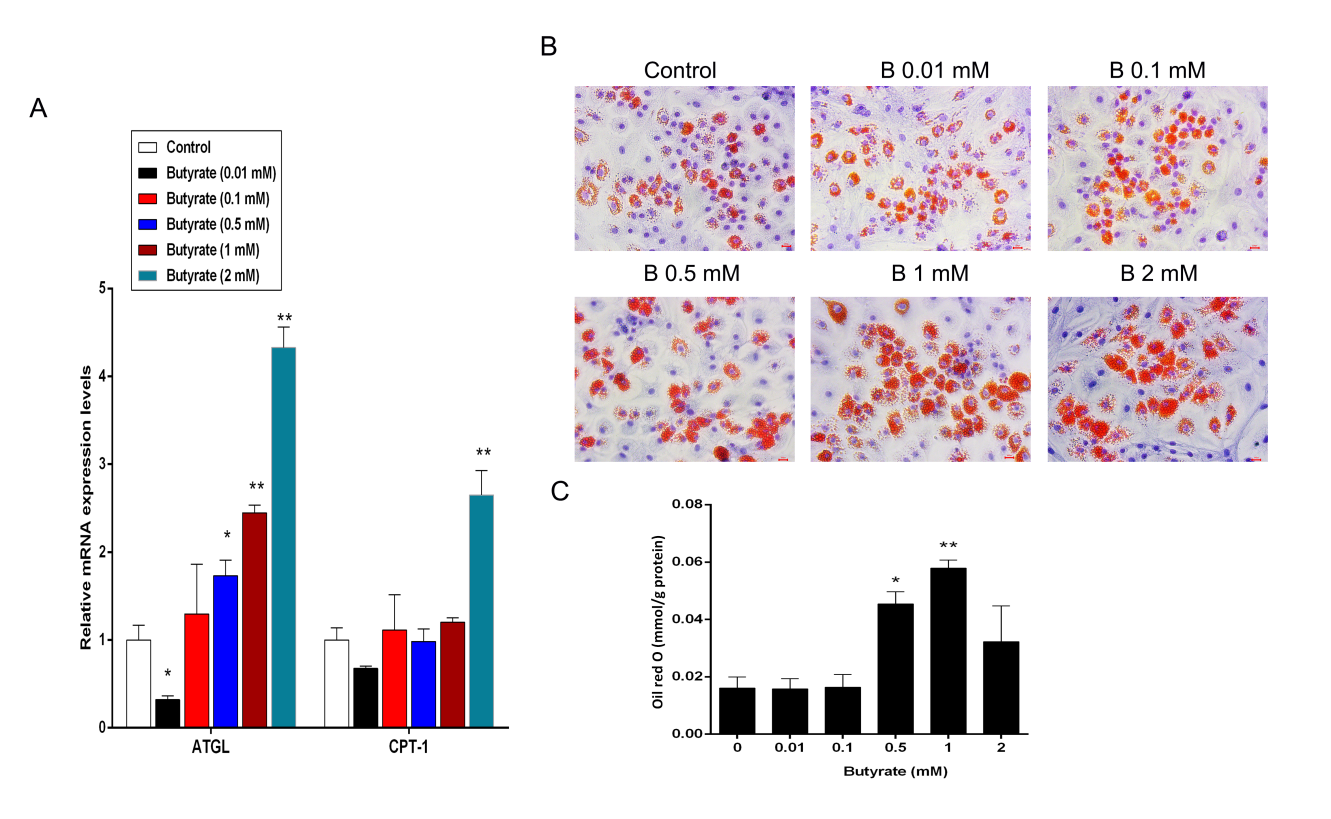


**Supplementary Figure 2. Effect of serial concentrations of SB on lipid metabolism of differentiation adipocytes.** The mature adipocytes were treated with serial concentrations of SB for 2 days. **(A)** Relative mRNA levels of ATGL and CPT-1 were determined by qRT-PCR in the treated cells (n=4). GAPDH serves as a control. **(B)** Lipid droplets visualized in the treated cells upon oil red O (red)-staining. The nuclei were stained with hematoxylin (purple). **(C)** The stained oil red O was extracted by isopropanol and quantitated, which was shown as mmol/g protein. **p*<0.05, ***p*<0.01 *vs.* the control by *t*-test.


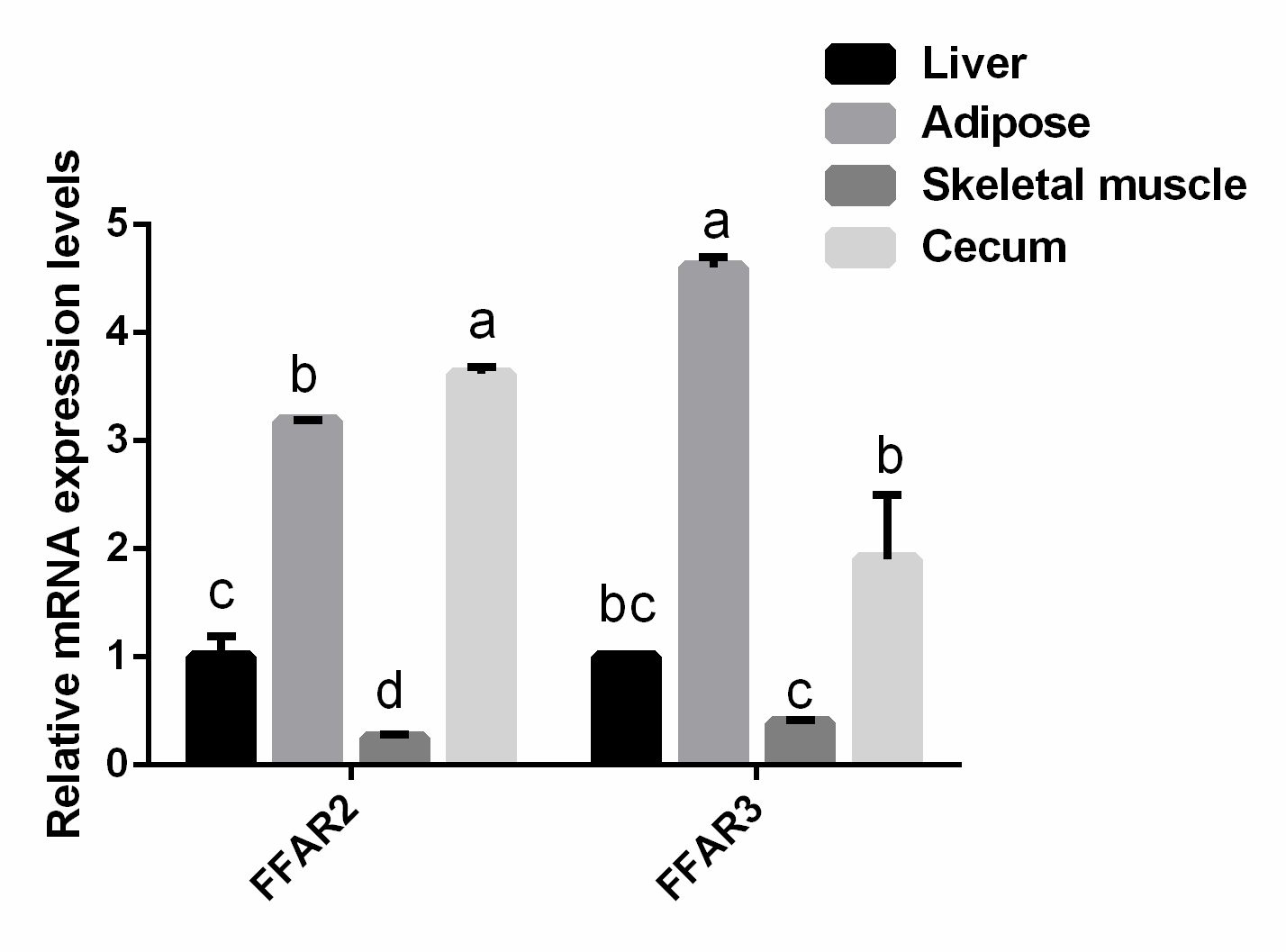


**Supplementary Figure 3. Relative FFAR2 and FFAR3 mRNA expression levels in different tissues from 19-day-old chicken embryos (n=6).** qRT-PCR analysis of FFAR2 and FFAR3 expression in different chicken tissues. The data are the means ± SEM (n=5). a-b means with different letters differ significantly.


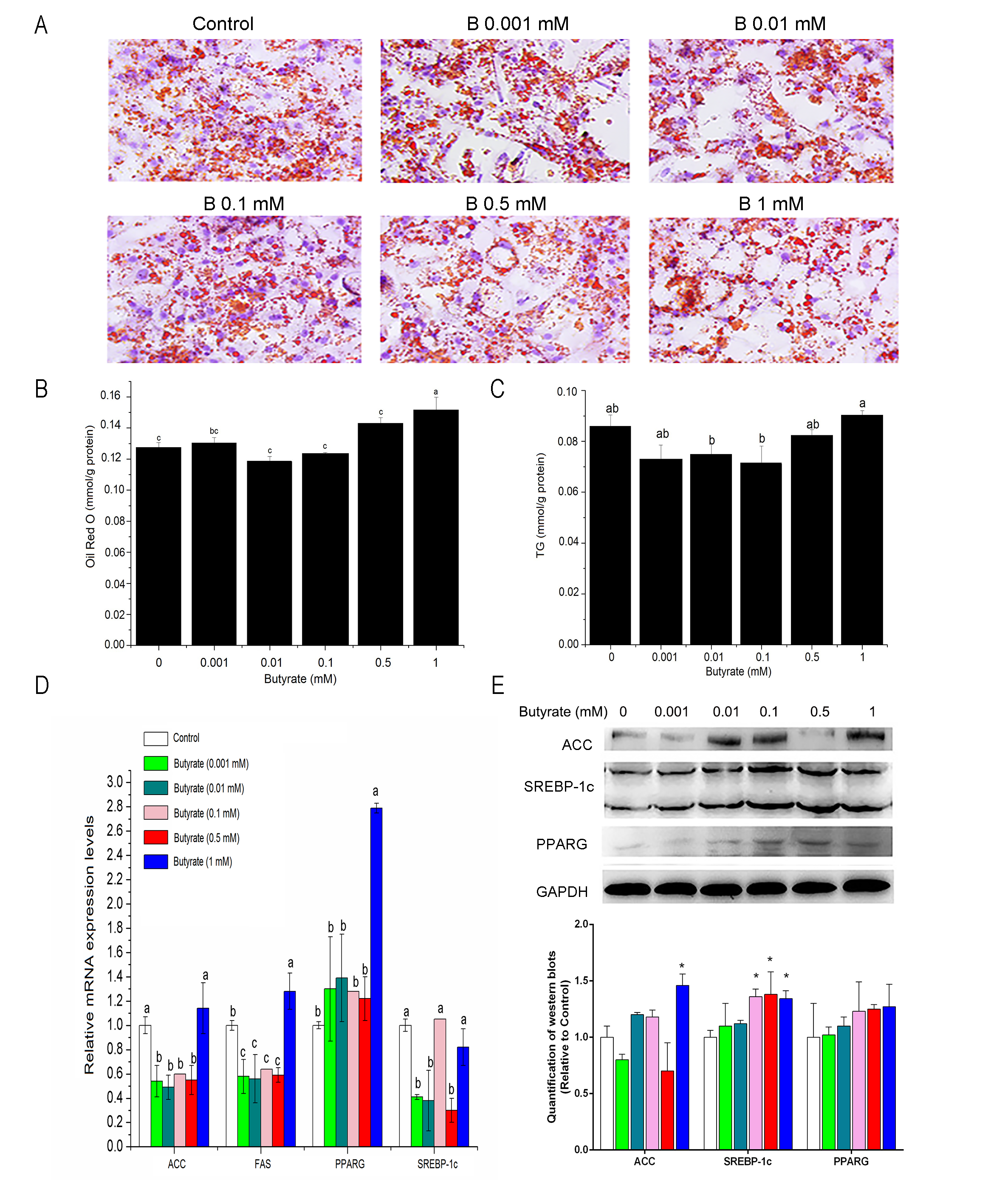


**Supplementary Figure 4. Effect of serial concentrations of SB on hepatocytic fat deposition.** Hepatocytes were treated with 0, 0.001, 0.01, 0.1, 0.5 and 1 mM SB for 3 days. **(A)** Oil red O staining (red) was conducted in hepatocytes treated with different SB concentrations. The nuclei were stained with hematoxylin (purple). **(B)** The stained oil red O was extracted by isopropanol and quantitated, which was shown as mmol/g protein. **(C)** Quantification of the accumulated TG based on the same protein content. **(D)** Relative mRNA levels of lipogenic markers determined by qRT-PCR in the treated cells. The mRNA levels were normalized to GAPDH. **(E)** Western blot images and quantification analysis showing expression of lipogenic markers on day 3 post-treatment (n=3). GAPDH was used as an internal control. The values are the means ± SEM (n=3–6). ^a-b^means with different letters differ significantly.


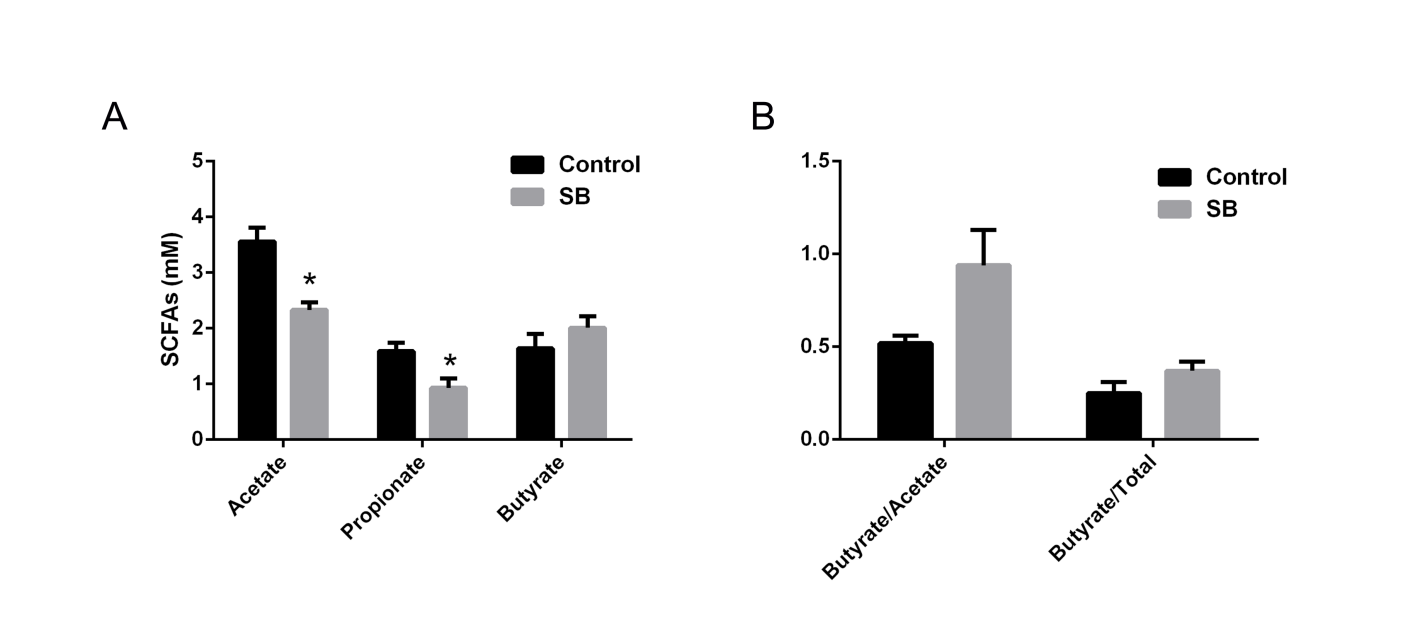


**Supplementary Figure 5. The SCFA contents in the cecal chyme in control- and SB-treated broilers at d 21.** **(A)** Acetate, propionate and butyrate concentrations in the cecal chyme of broilers. **(B)** Butyrate/acetate and butyrate/total SCFAs. SCFA concentrations in the cecal chyme were tested by GC−MS. The data are the means ± SEM (n=5). ^*^ *p*< 0.05 *vs.* the control.

**
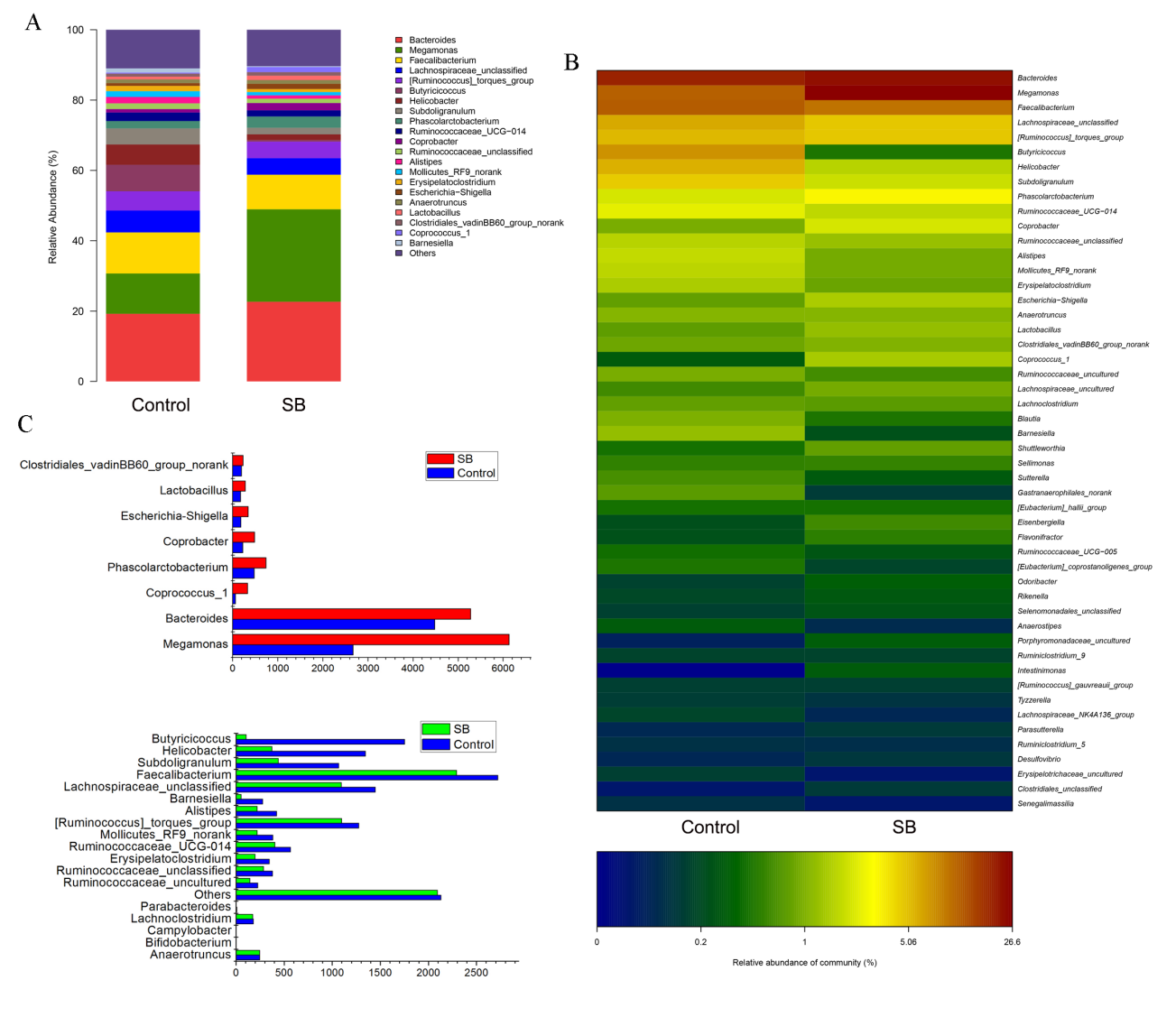
**

**Supplementary Figure 6. Alteration of the cecal microbiome structure by dietary SB supplementation. (A)** The gut microbiota structure at the genus level. **(B)** Heatmap analysis of the gut microbiota in the control and SB-treated broilers at genus level. The heat map visualizes the abundance of the gut microbiome in the two groups (n=6). **(C)** Comparison of the largely changed bacteria at the genus level (n=6). The red and green pillars indicate the increased and decreased genera in the treatment group, respectively. The blue pillars indicate the genera in the controls.

**
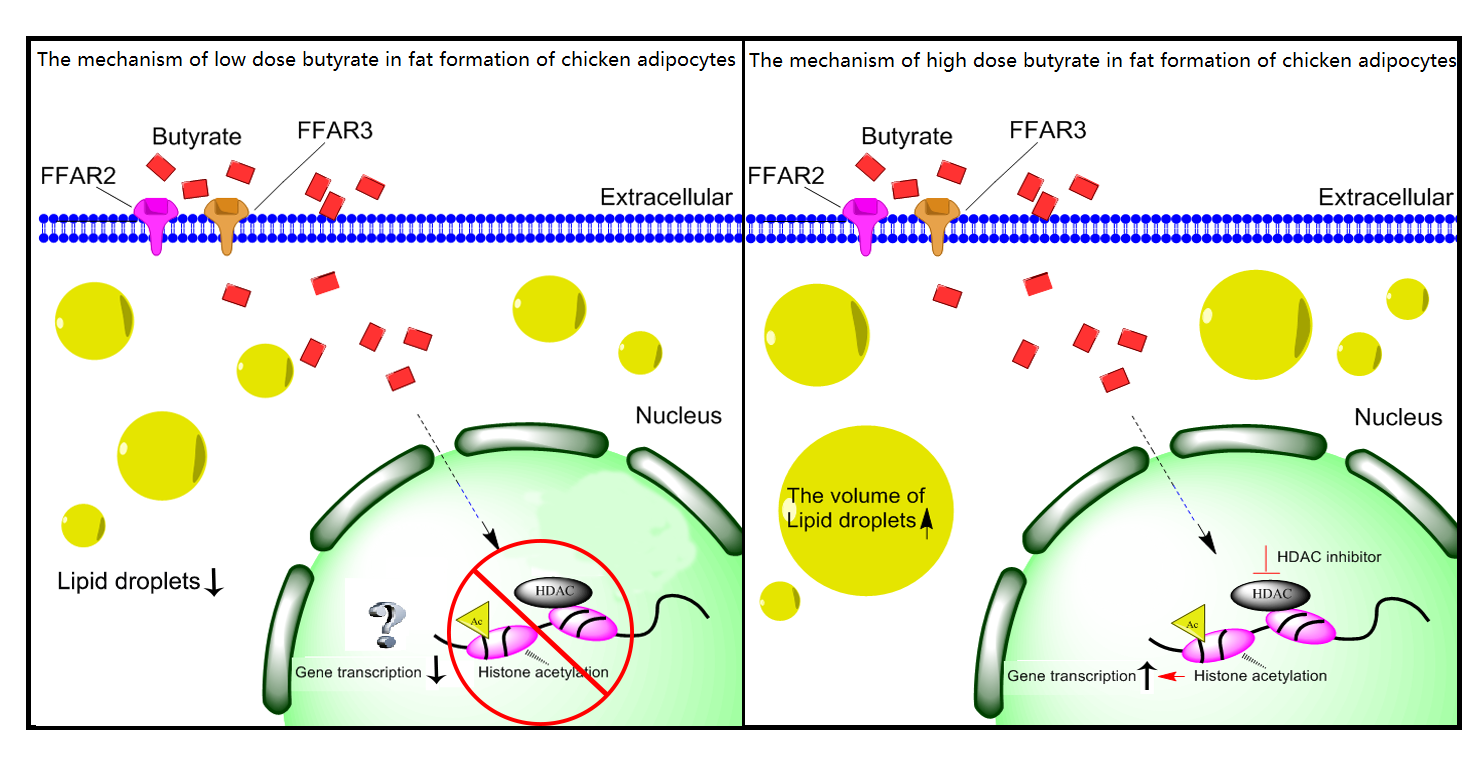
**

**Supplementary Figure 7. A model of the difference of low and high concentrations of butyrate on fat accumulation in chicken adipocytes. (A)** Physiological low dose butyrate inhibits adipogenic genes expression and adipocytic fat accumulation partially via FFARs. **(B)** High dose butyrate stimulates adipogenic genes expression and enlarges fat droplets in chicken adipocytes partially via inhibiting HDAC activity.

**Supplementary Table 1.** The composition and nutrient levels of the experimental diets (0−42 d).

| **Ingredients (%)** | **0-21d** |  | **21-42d** |
| --- | --- | --- | --- |
| Corn (8.5% CP) | 52.05 |  | 54.66 |
| Soybean meal (43% CP) | 38.02 |  | 32.81 |
| soybean oil | 5.79 |  | 8.5 |
| Mountain flour | 0.99 |  | 1.13 |
| Dicalcium phosphate | 2.02 |  | 1.87 |
| NaCl | 0.32 |  | 0.29 |
| L-Lysine·H_2_SO_4_ (99%) | 0.1 |  | 0.12 |
| DL-Methionine (98%) | 0.2 |  | 0.18 |
| Choline chloride (50%) | 0.26 |  | 0.2 |
| Vitamin premix^*^ | 0.05 |  | 0.05 |
| Mineral premix^†^ | 0.20 |  | 0.20 |
| **Calculated nutrient composition** | |  |  |
| Metabolizable energy (kcal/kg) | 3100 |  | 3300 |
| Crude protein % | 21 |  | 19 |
| Lys, % | 1.2 |  | 1.096 |
| Met, % | 0.48 |  | 0.44 |
| Met+Cys, % | 0.791 |  | 0.728 |
| Ca, % | 0.9 |  | 0.9 |
| Non-phytate P, % | 0.45 |  | 0.42 |
| L-Threonine % | 0.828 |  | 0.744 |
| Tryptophan % | 0.264 |  | 0.235 |
| Leucine % | 1.547 |  | 1.432 |
| Isoleucine % | 0.841 |  | 0.75 |
| Valine % | 0.996 |  | 0.897 |
| Crude fat % | 8 |  | 10.7 |

*Vitamin premix provides the following per kg of diet: VA, 8000 IU; VD_3_, 3000 IU; VE, 20 IU; VK, 2mg; VB1, 4mg; riboflavin, 8 mg; *D*-pantothenic acid, 11 mg; VB5, 40 mg; VB6, 4 mg; VB12, 0.02 mg; biotin, 0.15 mg; folic acid, 1.0 mg; choline, 700 mg

†Mineral premix provides the following per kg of diet: Fe (as ferrous sulfate), 80 mg; Zn (as zinc sulfate), 75 mg; Mn (as manganese sulfate), 80 mg; Cu (as copper sulfate) 10 mg, I (as potassium iodide), 0.40 mg; and Se (as sodium selenite), 0.30 mg.
